# Exploring Structure–Property Relationships of GAGs to Tailor ECM-Mimicking Hydrogels

**DOI:** 10.3390/polym10121376

**Published:** 2018-12-11

**Authors:** Ralf Zimmermann, Carsten Werner, James Sterling

**Affiliations:** 1 Leibniz Institute of Polymer Research Dresden, Max Bergmann Center of Biomaterials Dresden, Hohe Strasse 6, 01069 Dresden, Germany; werner@ipfdd.de; 2 Technische Universität Dresden, Center for Regenerative Therapies Dresden, Tatzberg 47, 01307 Dresden, Germany; 3 Riggs School of Applied Life Sciences, Keck Graduate Institute, 535 Watson Drive, Claremont, CA 91711, USA; jim_sterling@kgi.edu

**Keywords:** glycosaminoglycans, hydrogels, charge, structure, mucosa, ion pairing, desulfation, morphogen binding

## Abstract

Glycosaminoglycans (GAGs) are a class of linear polysaccharides that are ubiquitous in the extracellular matrix (ECM) and on cell surfaces. Due to their key role in development, homeostasis, pathogenesis, and regeneration, GAGs are increasingly used in the design of ECM-mimicking hydrogels to stimulate tissue formation and regenerative processes via specifically orchestrated cell-instructive signals. These applications first and foremost build on the ability of GAGs to effectively bind, protect, and release morphogens. The specificity and strength of morphogen-GAG interactions are largely governed by the number and spatial distribution of negatively charged sulfate groups carried by GAGs. Herein, we summarize a mean-field approach to quantify the density of ionizable groups, GAG concentration, and cross-linking degree of GAG-containing hydrogels on the basis of microslit electrokinetic experiments. We further present and discuss a continuum model of mucosa that accounts for charge regulation by glycan-ion pairing in biological contexts and under conditions of macromolecular crowding. Finally, we discuss the modulation of the morphogen binding and transport in GAG hydrogels by selective desulfation of the GAG component.

**Foreword:** On May 23, 2018, Paul Dubin, Professor of Chemistry at the University of Massachusetts Amherst, passed away at the age of 77 years. Paul dedicated many years of his scientific life to increase our understanding of structure–property relations of glycosaminoglycans. Towards this aim, he combined principles and methodologies of the life sciences and the physical sciences, particularly from the field of polyelectrolytes [1,2,3,4,5]. Because of his significant contributions to the field, Paul was an internationally respected expert, author of a many scientific reports, and invited speaker at scientific conferences. It was a privilege for us to welcome him at the 12th International Symposium on Electrokinetics (ELKIN 2017), held from September 10–12, 2017 at the Leibniz Institute of Polymer Research Dresden, Germany. With his keynote lecture “Heparin and heparan sulfates: The polyelectrolytes that aren’t”, he contributed to inspiring discussions and helped creating bridges between different approaches and fields of science. With this article, we would like to express our deep appreciation of his scientific achievements and our gratitude for his contribution to the success of the ELKIN 2017 conference.

## 1. Introduction

Glycosaminoglycans (GAGs) constitute an important component of the extracellular matrix (ECM) [1,6]. They are ubiquitous on cell surfaces and in connective tissues and control development, homeostasis, pathogenesis, and regeneration [6,7,8,9]. GAGs are flexible linear polysaccharides composed of repeating disaccharides that are heterogeneously decorated with sulfate and carboxylate groups [1]. The GAG family comprises the following members: hyaluronan, heparan sulfate, including the highly sulfated form heparin, chondroitin sulfate, dermatan sulfate, and keratan sulfate [9,10]. The chain length and sulfation patterns of GAGs vary over a broad range, depending on the tissue, age, and health condition of the organism [1,11]. The diversity arising from the structural heterogeneity is a consequence of the non-template driven biosynthesis which is nevertheless regulated to allow for modifications of GAG structures in response to cell and tissue development, as well as other exogenous and endogenous signals until today only partially understood [1].

GAGs are increasingly used for the design of ECM-mimicking hydrogels to stimulate tissue formation and regeneration via specifically orchestrated cell-instructive signals [11]. These applications build on the ability of GAGs to effectively bind, protect, and release morphogens. The specificity and strength of morphogen-GAG interactions are largely governed by the number and positions of negatively charged sulfate groups along the molecules. Based on that, the incorporation of different GAG densities or GAGs with graduated sulfation pattern into hydrogels enabled the development of materials to control cell fate decisions via cell-instructive morphogen gradients [12] as well as to modulate the spatio-temporal bioavailability of factors via affinity-based delivery [13] and sequestration [14,15] systems.

Beyond advanced synthesis and design concepts, progress in the development of novel materials for tissue engineering critically depends on the comprehensive analysis of the physico-chemical properties of the building blocks and their interactions with biologically active molecules as well as of the obtained molecular structures. Since GAGs give rise to strong electrostatic interactions this has to comprise the evaluation of the GAG and gel charge in aqueous environments as well as the analysis of charge-induced structural features and of electrostatic effects in the interactions of GAGs with various signaling molecules. In addition, the analysis of the cross-linking degree of the polymer networks and the resulting mechanical properties is of importance as it determines cellular response pattern [16] and the accessibility of the gels for target molecules.

Herein, we review recent physico- and biochemical approaches for the analysis of the charge formation, ion-specific charge regulation, and morphogen administration in GAG hydrogels in the context of the development of ECM and tissue models. Specifically, we present and discuss in Section 2.1 a mean-field approach to determine the charge, GAG concentration, and cross-linking degree in GAG hydrogels on the basis of microslit electrokinetic experiments. The net charge carried by the GAGs (and thus the strength and specificity of their interactions with morphogens) is further regulated by the pattern of local charge compensation by mono- or multivalent electrolyte ions. The impact of specific ion binding to ionized groups on the charge density in GAG-containing materials (e.g., hydrogel or mucosa) can be analyzed applying the continuum model presented in Section 2.2. In living organisms, the sulfation patterns of GAGs are enzymatically controlled in cells associated with the specific tissue. Building on rational design and synthesis concepts, this principle can be adapted for the design of cell-instructive hydrogels. In Section 2.3 we review and discuss an approach for the modulation of the morphogen administration in GAG hydrogels by selective desulfation of the GAG component.

## 2. Charge, Structure, Ion Binding, and Morphogen Transport in GAG Hydrogels—Case Studies

### 2.1. Charge and Structure of StarPEG-heparin Hydrogels

The application of GAGs in ECM-mimicking hydrogels involves the formation of bulk and interfacial charges that, in turn, govern the structure and function of the materials and their interfaces as well as their interactions with (bio)molecules from the aqueous phase. Therefore, revealing the fundamental principles of interfacial and bulk charge formation and related phenomena is of utmost importance for optimizing the performance of biomaterials in demanding products and technologies such as bioactive cell culture scaffolds or biomolecular interaction analysis [17]. Stimulated by the growing interest of the scientific community in complex systems and the emerging fields of matrix engineering and lab-on-a-chip technologies, advanced experimental techniques and theories have been developed in recent years that facilitate the interpretation of charge-associated phenomena at a much higher level of sophistication [18,19,20,21].

**Figure 1:**
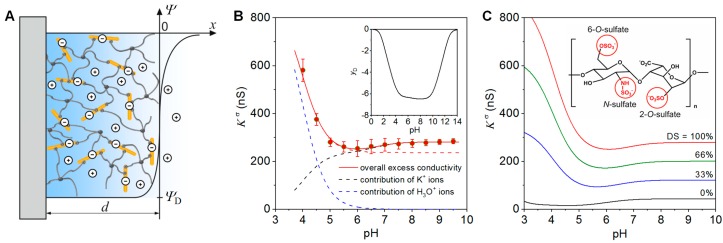
(**A**) Schematic representation of the model for the evaluation of surface conductivity data measured for starPEG-heparin hydrogel films. (**B**) Surface conductivity, *K*^σ^, and dimensionless Donnan potential, *y*_D_, of a starPEG-heparin hydrogel film with a thickness of ~600 nm in 0.1 mM KCl solution at varying pH values. The experimental data (red circles) were reproduced by the theory (red solid line) using the double layer model shown in (**A**). The dashed lines represent the contribution of K^+^ ions and H_3_O^+^ ions to *K*^σ^. (**C**) Simulation curves illustrating the impact of desulfation on the surface conductivity of starPEG-hydrogel films. For further details see text and Reference [22]. Reprinted from [22] with permission, copyright American Chemical Society, 2012.

Zimmermann et al. developed and applied a mean-field model to analyze the charge and structure of starPEG-heparin hydrogel films on the basis of surface conductivity data [22]. The hydrogel film was considered as 3D-meshwork where the ionization of sulfate and carboxylic acid groups along the heparin is related to an accumulation of counter ions that, in turn, leads to an elevated conductivity of the material and an electrical potential difference between the film and the electrolyte. Provided that the extension of the film in the direction normal to the surface, *d*, is much larger than the Debye screening length, interfacial effects are negligible. Under these conditions the potential difference between the hydrogel film and the bulk electrolyte can be described analogously to the potential difference across a semipermeable membrane by the Donnan potential (Figure 1A) [23]. In case of hydrogel films with a homogeneous distribution of the polymer segments and low to intermediate densities of ionisable groups, the surface conductivity, *K*^σ^, is related to the Donnan potential, *Ψ*_D_, between the hydrogel and an electrolyte according to the following equation [23]

(1) $$K^{\sigma }=\frac{F^{2}d}{RT}\sum_{i=1}^{N}z_{i}c_{i}D_{i}e^{-z_{i}y_{D}},$$

where *F* is the Faraday constant, *R* the gas constant, *T* the temperature, and *y*_D_ the dimensionless Donnan potential (*y*_D_ = *FΨ*_D_/*RT*). The electrolyte is considered to comprise *N* ions of valence *z_i_* with bulk concentrations *c_i_*, with the index *i* running from *i* = 1,…,*N*.

In Figure 1B we show the surface conductivity of a starPEG-heparin film with a thickness of ~600 nm at varying pH values of a 0.1 mM KCl solution. The film was prepared at a molecular ratio starPEG to heparin of 3, i.e., in the case of a quantitative reaction of the starPEG, a conversion of 12 of the 24 carboxyl groups of the heparin would occur [24]. The surface conductivity provided detailed insights into the ionization of the sulfate and carboxyl groups at the heparin in the hydrogel as well as the pH-dependent pattern of charge compensation. In the acidic pH range, *K*^σ^ is determined by the concentration and mobility of counter ions that neutralize the charge of the strongly acidic sulfate groups. The strong increase of *K*^σ^ below pH 5 results from the replacement of the K^+^ ions, which predominantly compensate for the gel charge at neutral and alkaline pH, by H_3_O^+^ ions at lower pH values (for the contributions of K^+^ and H_3_O^+^ ions to *K*^σ^ see black and blue dashed lines in Figure 1B). H_3_O^+^ ions have a ~5 times higher mobility than K^+^ ions [25], leading to the increase in *K*^σ^ observed. The absence of the less acidic carboxyl groups due to complete conversion of the heparin carboxyl groups during the gel formation, would result in a plateau of *K*^σ^ at neutral and weak alkaline pH (see red dashed line in Figure 1B). According to the molecular composition, 50% of the carboxyl groups were involved in the gel formation. Therefore, the increase of the surface conductivity above pH 6 unambiguously results from the ionization of non-converted carboxyl groups in the film [22]. As demonstrated below, this feature provides the possibility to derive information on the cross-linking degree in the hydrogel by measuring *K*^σ^ [22,24].

The mean-field model further allows us to determine the concentrations and p*K* values of ionizable groups in hydrogel film. The comparison of the experimental data with simulation results (Figure 1B) revealed 53 mmol/L and p*K* = 0.8 for the sulfate groups and 10 mmol/L and p*K* = 4 for the carboxyl groups [22]. As an important prerequisite for the further analysis of the gel composition and its cross-linking degree, heparin of known molecular weight and independently determined number of sulfate and carboxyl groups per molecule was used for the gel formation [22]. As the sulfate groups are not involved in the formation of the polymer meshwork [26], the concentration obtained from the fit of the experimental data can be used to estimate the heparin concentration within the hydrogel film. This calculation revealed a heparin concentration of ~11 µg/µL, which agrees well with values obtained for similar macroscopic starPEG-heparin gels [26]. The obtained heparin concentration can be used further to gain information on the real cross-linking degree of the hydrogel forming building blocks. For the hydrogel films discussed here, the analysis provided in excellent agreement with the expected value an average number of 12.3 carboxyl groups per heparin molecule.

As discussed in detail in Section 2.3, desulfation of heparin is an appropriate means to tune its interactions with signal molecules. Applying the mean-field approach [22], modification of the sulfation pattern of the GAG components in hydrogel films can be quantified. The variation of the surface conductivity with the degree of sulfation (DS) is shown in the simulation results given in Figure 1C. Compared to the fully sulfated heparin (red curve in Figure 1C), the chemical conversion of the 2-O-sulfate, 6-O-sulfate and/or N-sulfate groups [27] would cause significantly lower surface conductivity values. As desulfation results in a significant drop of the electrical potential within the hydrogel film, the ionization of the carboxyl groups that are not involved in the gel formation is shifted towards the acidic pH range in case of lower sulfate density.

In summary, analysis of surface conductivity data is a label-free approach to quantitatively determine concentration, degree of conversion, and ionization of GAG components within binary hydrogels. The developed methodology has advantages over the labeling of unreacted groups for the determination of the cross-linking degree, because it is not prone to non-quantitative turnover or non-specific side effects. The obtained parameters can be used to tailor multifunctional hydrogel matrices for applications in regenerative medicine and point-of-care diagnostics.

### 2.2. Mucosal Model with Glycan-Ion Pairing

The starPEG-heparin hydrogel adjacent to a saline solution depicted in Figure 1A can be considered to be an in vitro model of a mucosal surface or an epithelial cell surface glycocalyx adjacent to a surface liquid layer (e.g., tears, saliva, or airway surface liquid in the lungs). GAGs and mucins tethered to the cell surface are hundreds of nanometers to several microns in thickness and as anionic polysaccharides, they provide a barrier to negatively charged pathogens, and serve as an attractant to arginine-rich peptides, growth factors, and cytokines. Cohen and Varki describe the glycocalyx as forest with a glycan canopy and proteoglycan tree trunks [28] while Richter et al. describe various polymer brush conformations of GAG self-organisation [10]. As with the starPEG-heparin hydrogels above, the anionic nature of mucosa is largely governed by the degree of sulfation while the specific cations electrically neutralize the layer and establish a negative Donnan potential relative to the surface liquid. The degree of sulfation is controlled by genetic regulation of specific sulfotransferases and sulfatases in cells associated with specific tissues [29,30].

**Figure 2:**
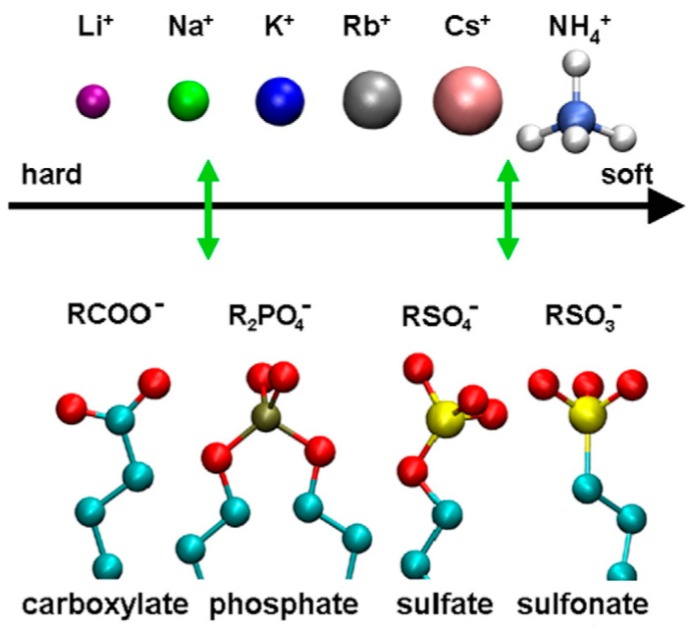
Affinity ordering of anionic surfactant headgroups and the respective counterions. Reprinted from [33] with permission, Copyright Elsevier, 2009.

In biological systems, ion-specific behavior, or lyotropy, is largely controlled by the nature of hydration of ions and results in a host of biophysical effects including the salting-in and salting-out of proteins, i.e., the Hofmeister effect. The biophysical basis of lyotropy can be characterized by the nature of potentials of mean force between charged groups in water, as a function of the distance between charge centers. Free energy minima are typically seen for the pairs that represent contact ion pairs and solvent separated ion pairs. A treatise on ion pairing is provided by Marcus and Hefter [31] and a volume on specific ion effects was edited by Kunz [32]. A graphical representation of favored Δ*G* pairing is shown by ordered pairing in Figure 2 from Vlachy et. al [33]. Quantitatively, the authors show that acetate prefers sodium to potassium by about 2.5 kcal/mol while methylsulfate favors potassium to sodium by about 0.4 kcal/mol.

These numbers can be used to estimate counterion binding in GAG hydrogels, but it is also important to emphasize that biological control of ion concentrations is tightly regulated and multiple types of cations, including divalent cations Mg^2+^ and Ca^2+^ are all present in most any living tissue in crowded macromolecular environments. Therefore, the thermodynamics of binding are not dilute bimolecular interactions that can be accurately quantified for in vivo conditions. In addition to screening and ion pairing of free cations, GAG-protein interactions utilize salt-bridges to lysine and arginine residues to affect binding. Although not represented among the cations in Figure 2, arginine occupies a privileged role in biology; ion pairing of arginine and sulfate has been characterized for its important role in growth factor binding to GAGs and in ion-exchange chromatography [34,35,36].

Sterling and Baker recently proposed an approach to model these ion-specific effects in glycan-rich environments to quantify counterion-condensation through the use of local stoichiometric dissociation constants, *K_ij_*, where *i* represents an ion and *j* a charged group on a fixed GAG polymer [37]. The formulation accommodates simultaneous pairing of all possible partners to the polymer fixed charges. The result is a local quasi-equilibrium giving the fixed polymer unbound fraction as

(2) $$\frac{c_{j}}{c_{j,o}}={\left[1+\overset{N}{\underset{i=1}{\sum }}\frac{c_{i}}{K_{ij}}\right]}^{-1}$$

Here, *c_j_* is the local concentration of unbound charges of type *j*, *c*_*j*,o_ is the local concentration of charges of type *j*, *c_i_* represent the *N* different types of free ions. From this equation, one can see that in a GAG-based hydrogel, the local unbound concentration of carboxylates (*j* = 1, for example) and sulfates (*j* = 2) is dependent on hydronium (*i* = 1, for example) and all of the other cations or cationic groups (*i* > 1). This formulation shows the importance of the dimensionless values given by the ratio of the local concentrations to the dissociation constants. Even for weak-pairing dissociation constants in the 1–100 millimolar range, ion concentrations in biology can be high enough that these effects can be substantial.

**Figure 3:**
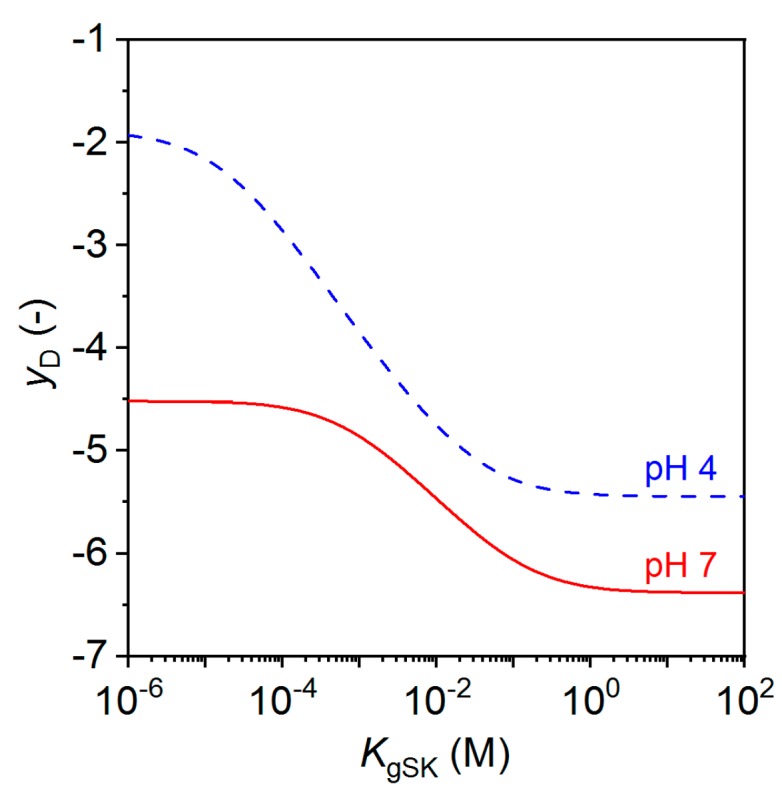
Dimensionless Donnan potential, *y*_D_, vs gel sulfate-potassium dissociation constant, *K*_gSK_, for different pH values. Reprinted from [37] with permission, Copyright 2018, Wiley-VCH Verlag GmbH & Co., 2018.

Boltzmann partitioning of free ions between a GAG layer and an adjacent liquid layer, in conjunction with electroneutrality, results in the establishment of a Donnan potential. With the simultaneous solution of partitioning and counterion binding to charged gel groups, the equilibrium established can be denoted electrolyotropic equilibrium. Applying this approach to the gel model in Figure 1B,C, the model can then address the pairing of sulfate groups with potassium using a local stoichiometric dissociation constant shown as *K*_gSK_. In dilute solutions, the pairing of sulfates to potassium would be expected to be quite weak—at least in the 1–10 molar range—but here it is considered a variable. Figure 3 shows that with other conditions held constant, stronger pairing (smaller *K*_gSK_) results in a smaller negative Donnan potential required to attract sufficient counterions for electroneutrality, and therefore a lower surface conductivity. For example, at a pH of 7, a dissociation constant above molar values requires *y*_D_ to achieve electroneutrality near −6.4 while a millimolar or smaller dissociation constant results in enough ion pairing to reduce *y*_D_ in magnitude to around −4.5. Note that both hydronium and potassium are included in the model with the result that lower pH also leads to smaller-magnitude *y*_D_ values.

The binding of counterions to anionic headgroups represented by Equation (2) can be applied more broadly than the two-layer model shown in Figure 1A. In combination with surface conductivity measurements, the model provides the possibility to determine stoichiometric binding/dissociation constants for ionic interactions at surfaces. In GAG-rich heterogeneous environments, including the ECM and soft-diffuse interfaces with spatially-varying charge densities, a Poisson–Boltzmann formulation can be amended to include ion binding. Although protonation of acidic molecules has often been included in such formulations [18,19,23], binding of other cations should also be taken into account when ion-exchange processes are significant.

Summarizing, electrolyotropic equilibrium across a gel-liquid interface can be considered as a structural model of GAG hydrogels or mucosal surfaces. The gel equilibrium would be expected to respond to a liquid salt composition change on the time scale of ion electrophoresis and ion-pairing reaction times. These equilibration processes occur very fast compared with the time of polyelectrolyte diffusion, polyectrolyte complexation, or GAG-protein interactions such as those presented in the following section.

### 2.3. Modulation of Morphogen Administration in StarPEG-heparin Hydrogels

**Figure 4:**
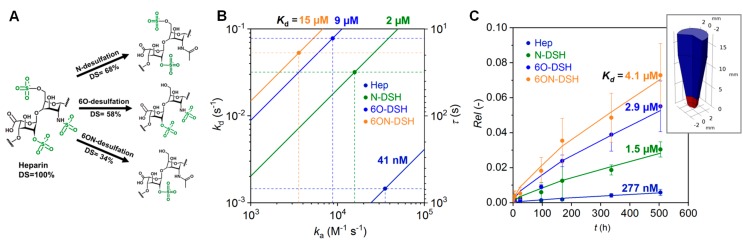
(**A**) Schematic representation of the selective removal of heparin’s sulfate groups at one or more positions resulting in different degrees of sulfation (DS). (**B**) Plot of dissociation rate constants, *k*_d_, vs the association rate constants, *k*_a_, for *K*_d_ values (*K*_D_ = *k*_d_/*k*_a_) obtained from biolayer interferometry (BLI) for the interaction of PDGF-BB with the heparin derivatives. In combination with the dashed lines, the circles indicate the rate constants *k*_d_ and *k*_a_ determined via BLI. The solid lines represent all possible combinations of *k*_a_ and *k*_d_ associated with the given *K*_d_ values. The parameter *τ* is the residence time, characterizing the life time of the PDGF-BB-heparin complexes. (**C**) Release of PDGF-BB from hydrogels formed from starPEG and heparin derivatives. Circles represent experimental data and the solid lines release profiles calculated on the basis of a reaction-diffusion model. The release experiments were performed in low protein binding tubes, see inset. The volume shown in red represents the hydrogels and the volume shown in blue the release medium. Adapted from [12] with permission, Copyright Elsevier, 2018.

The development of tissues and organs is largely controlled by the spatio-temporal cues presented by the ECM to embedded cells [38,39,40]. Therefore, the administration of morphogens is critically important for engineering ECM-mimicking hydrogels. Atallah et al. recently demonstrated how the morphogen transport and release from starPEG-heparin hydrogels can be tuned by selective desulfation of the heparin component. Heparin derivatives were obtained by desulfation at the N- (N-DSH), 6-O- (6O-DSH), or both the 6-O- and the 6-O-N- (6ON-DSH) positions (Figure 4A) and functionalized with maleimide moieties to allow for click reaction with thiol-functionalized starPEG or starPEG-peptide conjugates to form hydrogels in presence of protein-containing biofluids and cells [12].

The interaction of the platelet-derived growth factor-BB (PDGF-BB) with immobilized heparin derivatives was studied using biolayer interferometry (BLI). The experiments revealed significant differences in the affinity of PDGF-BB to the selectively desulfated heparins (Figure 4B). With a dissociation constant of *K*_d_ ~ 41 nM, the highest affinity was found for the native heparin. The affinity of PDGF-BB to the heparin derivatives decreased in the order Hep > N-DSH > 6O-DSH > 6ON-DSH. For the 6ON-desulfated heparin the affinity was found to be about three orders of magnitude lower (*K*_d_ ~ 15 µM). The residence time *τ* (*τ* = *k*_d_^−1^ where *k*_d_ is the dissociation rate constant derived via BLI), characterizing the life time of the PDGD-BB-heparin complexes, and the corresponding half-life time of the complexes (*τ*_1/2_ = −ln(0.5)/*k*_d_) changed about two orders of magnitude (Figure 4B) [41].

To evaluate morphogen administration in 3-dimensional matrices, the release of PDGF-BB from starPEG-heparin hydrogels made of the different derivatives was studied. The hydrogels were prepared at the bottom of Eppendorf tubes and the release in the supernatant was analyzed for different time points using an enzyme-linked immunosorbent assay (Figure 4C). Using the binding constants obtained via BLI as starting points, the release data were reproduced with a numerical reaction-diffusion model taking into account steric interaction and binding in the gel. The calculated release profiles confirmed the release kinetics for the hydrogels formed out of the different heparin derivatives with a maximum of 7% PDGF-BB released for 6ON-DSH hydrogel. The dissociation constants for the PDGF-BB-heparin complexes derived from the fit of the data were in the same order of magnitude as the *K*_d_ values obtained from BLI (Figure 4C) which is in agreement within the variability range of *K*_d_ values when utilizing different determination methods [42,43]. Moreover, following the rational design concept for the hydrogels as morphogen delivery systems [11,13], the adjustment of the input parameters in the reaction-diffusion model such as the growth factor loading amount, the degree of sulfation, the ratio of the hydrogel volume to the release medium volume, provides the basis for the prediction of the release kinetics and morphogen gradient formation in in vitro and in vivo scenarios. Atallah et al. impressively used this option to direct the migration of morphogenesis of human mesenchymal stem cells [12]. Altogether, the modulation of the heparin charge by selective desulfation allows for the precise tuning of morphogen patterns in complex in vitro models involving multiple factors and different cell types as previously established to modulate signaling in angiogenesis [44,45] and to induce human renal tubulogenesis [46]. With respect to the central role of GAGs in developmental processes, homeostasis, pathogenesis, and regeneration [6,7,8], tailored GAG hydrogels provide a base to gain new insights into these processes from in vitro models.

## 3. Conclusions and Perspectives

To capitalize on the central role of GAGs in living matter for the development of novel biomaterials, advanced synthesis and design concepts as well as a comprehensive analysis of the physico-chemical properties of the building blocks, their interactions with biologically active molecules, and of the obtained molecular structures are required. Electrokinetics in combination with advanced modelling of the electrohydrodynamics of soft materials provides valuable options for analyzing the interfacial charge and structure of GAG-based materials as well as their interactions with ions and (bio)molecules from the liquid phase. As a specific example, we report an approach for the quantification of the GAG concentration, degree of conversion, and of the sulfate and carboxyl content in GAG hydrogels. Since analytical approaches to determine these parameters via label-based methods are prone to non-quantitative turnover or non-specific side effects, the sensitive and quantitative method is clearly advantageous for the development of GAG-based materials. We further present and discuss a Poisson–Boltzmann model that incorporates lyotropic effects in the glycocalyx and in mucin layers. The model provides a basis to analyze ion binding to charged groups carried by GAGs and to correlate the resulting net charge densities with the strength and specificity of their interactions with morphogens. Finally, we demonstrate how the morphogen transport within and release from starPEG-heparin hydrogels can be tuned by selective desulfation. This approach offers unprecedented options for controlling the spatio-temporal concentration of signal molecules in in vitro tissue and disease models [12]. Future developments will have to aim at a better understanding of structure–property relations of GAGs at the molecular level. This necessarily requires the combination of principles and methodologies of the life sciences and the physical sciences. With his pioneering work [1,2,3,4,5], Paul Dubin provided fundamental new insights in this complex world.
